# Enhancing low-light images using Sakaguchi type function and Gegenbauer polynomial

**DOI:** 10.1038/s41598-024-80605-w

**Published:** 2024-11-29

**Authors:** K. Sivagami Sundari, B. Srutha Keerthi

**Affiliations:** Department of Mathematics, School of Advanced Sciences, Vellore Institute of Technology, Chennai Campus, Chennai, 600127 India

**Keywords:** Applied mathematics, Computer science

## Abstract

Enhancing low-light images is crucial for various applications in computer vision, yet current approaches often fall short in balancing image quality and detail preservation. This study introduces a novel method designed to enhance low-light images by applying advanced mathematical techniques from geometric function theory. Specifically, we employ Sakaguchi-type class functions, subordinated with the Gegenbeur polynomial, to derive coefficient estimations. These estimations are then used in convolution kernels to produce enhanced image versions. The method was tested on the LOw-Light dataset (LOL), containing challenging low-light images with noise and artifacts. Our approach’s effectiveness is validated through quantitative metrics, including PSNR and SSIM, as well as visual comparisons. The results demonstrate significant improvements over existing state-of-the-art methods, offering better visibility and detail retention. This method holds promise for enhancing images in critical fields such as surveillance and medical imaging.

## Introduction

Advancements in technology and photographic equipment have increased the demand for high-quality images. However, environmental factors often hinder the acquisition of desired images, resulting in issues such as blurred details, uneven lighting, low light conditions, and backlighting that affect image quality. To tackle these challenges, low-light image enhancement techniques have become instrumental. These techniques are of immense importance in fields such as computer vision (for tasks like target detection and recognition), surveillance systems, home security, medical image segmentation, and autonomous driving. Notably, many existing algorithms primarily focus on brightening images but often overlook the preservation of essential original image details. This often results in a reduction in the information entropy of the image, hindering the complete expression of the original image information.

Numerous algorithms have been proposed to address the challenge of enhancing low-light images, with the histogram equalization technique being a commonly employed method due to its avoidance of saturation and simplicity. However, this method has its limitations. It enhances low-light images by using the entire image’s histogram details as a transformation function, primarily focusing on improving contrast. This singular focus on contrast enhancement can result in over-enhancement in areas with low contrast, sometimes leading to regions that are either under or over-enhanced within the image.

Similarly, Wang et al.’s^1^ algorithm aimed to enhance contrast while preserving illumination in the image. Unfortunately, this approach fell short in maintaining the overall visual quality of the image. A different perspective can be found in techniques inspired by Retinex theory, originally developed to model human color perception, which has been effectively applied to enhance images captured under challenging lighting conditions. This theory posits that color perception is influenced by both the illumination and the reflectance properties of objects. While Retinex can theoretically facilitate image decomposition into illumination and reflectance, this is primarily effective under conditions of uniform lighting and for matte surfaces. Consequently, many contemporary techniques inspired by Retinex focus on estimating and removing the illumination component to enhance overall image quality. For example, Wang et al.^2^ proposed methods that focus on eliminating the illumination component, while others, such as the approach outlined in Wang et al.^1^, aim to retain a portion of the illumination to preserve natural image effects. However, these methods often introduce distortions that compromise visual quality, as they fail to account for the intrinsic characteristics of the camera’s response. Additionally, notable implementations of Retinex that do not perform image decomposition, such as SuPeR^3^, Light Random Spray Retinex^4^, STAR^5^, and Milano-Retinex^6^, provide alternative approaches to improving image clarity and detail.

Furthermore, certain techniques involve image dehazing, aiming to preserve the natural distribution of pixel values, as exemplified by the work of Dong et al.^7^, who proposed a method for dehazing low-visibility input images. Subsequently, image inversion is performed to obtain illuminated images. The dark channel prior, as proposed in Ref.^8^, is a well-known technique focused on enhancing the quality of hazy images characterized by low visibility. The colour attenuation prior^9^ has been presented as an effective approach for recovering deteriorated hazy images. Cai et al.^10^ investigated learning-based dehazing for an end-to-end haze removal procedure, while Ancuti et al.^11^ presented a fusion-based night picture dehazing approach. Many researchers have been working on ways to improve low-light images in various environments^12–15^. Although these methods yield satisfactory results, they may not fully capture the true illumination and contrast of the scene. Additionally, some of these approaches do not account for the impact of noise in resulting images, potentially leading to varying results under different lighting conditions.

In addition to the approaches discussed earlier, several related works have significantly contributed to the field of image enhancement. For instance, the study *“Bilateral Tone Mapping Scheme for Color Correction and Contrast Adjustment in Nearly Invisible Medical Images”*^16^ addresses the challenges of poor illumination in medical imaging. The authors propose a method that enhances contrast while maintaining natural color quality, effectively improving the visibility of crucial details in medical images.Another notable contribution is the work by Bhandari et al.^17^, their study emphasizes the importance of balancing contrast and brightness in color images, particularly under suboptimal lighting conditions. By optimizing these elements, the authors demonstrate improved color fidelity, making their technique valuable for applications where accurate color representation is essential. Furthermore, Subramani et al.^18^ introduced a method that effectively restores visual quality in degraded images through intensity mapping adjustments. By manipulating the Bezier curve, the authors were able to enhance contrast and reveal hidden details, making it a useful tool for improving image quality in various scenarios. In^19^, by applying an optimized Bezier curve-based intensity mapping scheme, the authors were able to enhance visibility and detail in dark images. This advancement is particularly relevant for applications such as night photography and surveillance, where capturing high-quality images under low-light conditions is crucial.

The application of geometric function theory in computer vision remains a relatively unexplored domain, with only a limited number of researchers venturing into this area. Notably, Priya et al.^20^ introduced the class $C_\Sigma$, utilizing a Sakaguchi type class subordinated with a Horadam polynomial, where the coefficient bounds of $C_\Sigma$ play a pivotal role in texture enhancement. Similarly, Nithiyanandham et al.^21^ defined a class $p-\Phi S^* (t,\mu ,\nu ,J,K)$, crafted from a Mittag-Leffler type Poisson distribution, and investigated its geometrical properties. By making use of the coefficient bounds derived from this class, Nithiyanandham et al.^22^ achieved successful enhancements in retinal images. Aarthy et al.^23^ further extended these findings by applying the same class with varied parameter values to enhance images from datasets like ’DAISY,’ ’MEDICAL,’ and ’MISCELLANEOUS.’ The coefficient bounds, serving as critical factors, influence the enhancement process by providing essential constraints.

Despite these contributions, a notable knowledge gap exists in understanding the coefficient bounds of Gegenbauer polynomials and their application in low light image enhancement. This study aims to address this gap by determining the coefficient bounds of Gegenbauer polynomials. Subsequently, the obtained coefficient bounds are convoluted in eight different directions using a $3 \times 3$ mask. This innovative approach facilitates uniform enhancement across the entire image. Our research contributes to advancing the understanding and application of geometric function theory in low-light image enhancement.

The structure of this article is as follows: Section “Mathematical interpretation” explains the mathematical interpretation of coefficient bounds. In “Proposed methodology”, we provide a detailed explanation of our proposed model. Sections “Experimental analysis and results” and “Performance analysis” address both qualitative and quantitative assessments, comparing our approach to other cutting-edge methodologies. Finally, “Conclusion” contains the conclusion of our study as well as a discussion on future research prospects.

## Mathematical interpretation

Gegenbauer polynomials, a set of orthogonal polynomials, have been applied across various domains, including image processing. In image processing, the moments derived from these polynomials are used for tasks such as image representation^24^ and analysis^25^. To achieve a controlled image enhancement process, we utilize the concept of coefficient bounds from geometric function theory. This requires the construction of a specific class, for which we employ the Gegenbauer polynomial and the Sakaguchi type function, combined through subordination. This subordination creates a mathematical connection between the Sakaguchi-type function and the Gegenbauer polynomial, enabling us to define a new class, denoted as $G_S (\Phi )$ . For defining the class and determining the coefficient bounds, we provide a few fundamental definitions below. The coefficient bounds derived from this new class are central to our enhancement process, as they control each element of the convolution kernel based on local image characteristics. This targeted use of coefficient bounds provides a structured and adaptable approach to enhance image clarity, particularly under challenging low-light conditions.

The class of all *analytic functions* of the form

1 $$\begin{aligned} f(\mu )=\mu +\sum _{(n=2)}^\infty a_n \mu ^n \end{aligned}$$

is denoted by ${\mathcal {A}}$ and normalized in the open unit disk ${\mathscr {D}} = \{ \mu :|\mu |<1 \}$. The aforementioned equation is then substituted in the class $G_S (\Phi )$ to derive the coefficient bounds. ${\mathcal {S}}$ is the class of all $univalent \ function$ (one-to-one) in ${\mathcal {A}}$. According to the *Koebe one-quarter theorem*, the image of ${\mathscr {D}}$ under every univalent function $f \in {\mathscr {A}}$ contains a disk of radius $\frac{1}{4}$ and it states that for every univalent function $f \in {\mathscr {A}}$ there exits a inverse map $f^{-1}=g$ satisfying

$$\begin{aligned} f^{-1}\{f(\mu )\} = \mu \ and \ f\{f^{-1}(\mu )\} = \mu , \ \mu \in {\mathscr {D}}, \ |\mu |<\frac{1}{4}. \end{aligned}$$

and

2 $$\begin{aligned} f^{-1}(\nu ) = \nu - a_2\nu ^2+(2a_2^2-a_3)\nu ^3-(5a_2^3-5a_2a_3+a_4)\nu ^4+\cdots = g \end{aligned}$$

is the inverse function. An analytic function ${\mathcal {A}}$ in ${\mathscr {D}}$ is said to be $bi-univalent$ if both $f,f^{-1}$ are univalent in ${\mathscr {D}}$. Let $\Sigma$ be the class of all bi-univalent functions in the unit disk ${\mathscr {D}}$. Regarding early results on analytic and bi-univalent functions, refer to Refs.^26,27^.

Let the two functions $f, \ g$ be analytic in ${\mathscr {D}}$ and $\varpi$ be a schwarz function satisfying $\varpi (0)=0$ and $|\varpi (\mu )| < 1$ such that $f(\mu )=g(\varpi (\mu ))$ then *f* is said to be *subordinate* to *g*, or $f \prec g$. If *g* is univalent, then $f \prec g$ iff $f(0)=g(0)$ and $f({\mathscr {D}}) \subset g({\mathscr {D}})$. The generating function of $Gegenbauer \ polynomials$ is defined by

3 $$\begin{aligned} H_{\Phi }(x,\mu )=\frac{1}{(1-2x\mu +\mu ^2)^{\Phi }} = \sum _{n=0}^{\infty } C_n^{\Phi }(x)\mu ^n , \end{aligned}$$

where $\Phi$ is a nonzero real constant, $x \in [-1,1]$ and $\mu \in {\mathscr {D}}$. Gegenbauer polynomial satisfies the below recurrence relations:

4 $$\begin{aligned} C_{n}^{\Phi }(x)=\frac{1}{n}[2x(n+\Phi -1)C_{n-1}^{\Phi }(x)-(n+2\Phi -2)C_{n-1}^{\Phi }(x)] \end{aligned}$$

with the initial values

5 $$\begin{aligned} C_0^{\Phi }(x)=1, \ C_1^{\Phi }(x)=2\Phi x , \ C_2^{\Phi }(x)=2\Phi (1+\Phi )x^2-\Phi . \end{aligned}$$

Special Cases:

1. When $\Phi =1/2$, then $C_{n}^{\Phi }(x)$ reduce to the Legendre polynomials.
2. When $\Phi =1$, then $C_{n}^{\Phi }(x)$ reduce to the Chebyshev polynomials.

Over the recent years, a growing number of researchers have been investigating the realm of bi-univalent functions in associated with orthogonal polynomials^28–31^.

### Class definition

A function $f\in \Sigma$ given by Eq. (1) is said to be in the class $G_S(\Phi )$ if the following conditions hold for all $\mu ,\nu \in {\mathscr {D}}$:

6 $$\begin{aligned} \frac{(1-t)\mu f' (\mu )}{f(\mu )-f(t\mu )} \prec H_{\Phi }(x,\mu ) \end{aligned}$$

and

7 $$\begin{aligned} \frac{(1-t)\nu g' (\nu )}{g(\nu )-g(t\nu )} \prec H_{\Phi }(x,\nu ), \end{aligned}$$

where $x\in (\frac{1}{2},1], -1 \le t < 1$, the function $g(\nu )=f^{-1}(\nu )$ is defined by Eq. (2) and $H_{\Phi }$ is the generating function of the Gegenbauer polynomial given by Eq. (3)

### Theorem

Let the function $f \in \Sigma$ given by Eq. (1) be in the class $G_S(\Phi )$. Then

8 $$\begin{aligned} |a_1|= 1 \end{aligned}$$

9 $$\begin{aligned} \small {|a_2| \le \frac{2|\Phi |x \sqrt{2|\Phi |x}}{\sqrt{|\Phi (2-u_2)^2- 2\tau x^2|}} } \end{aligned}$$

10 $$\begin{aligned} |a_3| \le \frac{4\Phi ^2 x^2}{(2-u_2)^2}+\frac{2|\Phi |x}{(3-u_3)} \end{aligned}$$

where $$u_n = \frac{1-t^n}{1+t}$$ , $$\tau = \Phi (1+\Phi )(2-u_2)^2-2\Phi ^2 [(3-u_3)-u_2(2-u_2)]$$

#### Proof

Let $f\in G_S(\Phi )$ then by definition (2.1) , we have

11 $$\begin{aligned} \frac{(1-t)\mu f' (\mu )}{f(\mu )-f(t\mu )} = H_{\Phi }(x,w(\mu )) \end{aligned}$$

and

12 $$\begin{aligned} \frac{(1-t)\nu g' (\nu )}{g(\nu )-g(t\nu )}= H_{\Phi }(x,v(\nu )), \end{aligned}$$

for some analytic functions

13 $$\begin{aligned} w(\mu )=c_1 \mu +c_2 \mu ^2 + c_3 \mu ^3+\cdots \ \ \ (\mu \in {\mathscr {D}}) \end{aligned}$$

14 $$\begin{aligned} v(\nu )=c_1 \nu +c_2 \nu ^2 + c_3 \nu ^3+\cdots \ \ \ (\nu \in {\mathscr {D}}) \end{aligned}$$

such that $w(0)=v(0)=0, |w(\mu )|<1 \ (\mu \in {\mathscr {D}})$ and $|w(\nu )|<1 \ (\nu \in {\mathscr {D}})$. It follows from Eqs. (11) and (12) that

15 $$\begin{aligned} \frac{(1-t)\mu f'(\mu )}{f(\mu )-f(t\mu )} = 1+C_1^{\Phi }c_1\mu +\bigg [C_1^{\Phi }(x)c_2+C_2^{\Phi }(x)c_1^2 \bigg ]\mu ^2+\cdots \end{aligned}$$

16 $$\begin{aligned} \frac{(1-t)\nu g'(\nu )}{g(\nu )-g(t\nu )} = 1+C_1^{\Phi }d_1\nu +\bigg [C_1^{\Phi }(x)d_2+C_2^{\Phi }(x)d_1^2 \bigg ]\nu ^2+\cdots \end{aligned}$$

By expanding (Eq. 1), we get

$$\begin{aligned} f(\mu ) = \mu +a_2 \mu ^2+a_3 \mu ^3+\cdots \\ f'(\mu )= 1+2a_2\mu +3a_3\mu ^2+\cdots \\ f(t\mu )= t\mu +a_2 t\mu ^2+a_3 t\mu ^3+\cdots \end{aligned}$$

Apply the aforementioned equations in (15)

17 $$\begin{aligned} \begin{aligned} (1-t)\mu [1+2a_2\mu +3a_3\mu ^2+\cdots ]= \bigg \{ 1+C_1^{\Phi }c_1\mu +\bigg [C_1^{\Phi }(x)c_2+C_2^{\Phi }(x)c_1^2 \bigg ]\mu ^2+\cdots \bigg \} \\ \bigg \{ \bigg [\mu +a_2 \mu ^2+a_3 \mu ^3+\cdots \bigg ] -\bigg [ t\mu +a_2 t\mu ^2+a_3 t\mu ^3+\cdots \bigg ] \bigg \} \end{aligned} \end{aligned}$$

By further expansion and equating the coefficient of $\mu ^2$ and $\mu ^3$, we arrive at the following equations

18 $$\begin{aligned} (2-u_2)a_2 = C_1^{\Phi }(x)c_1, \end{aligned}$$

19 $$\begin{aligned} (3-u_3)a_3-(2-u_2)u_2a_2^2 = C_1^{\Phi }(x)c_2+C_2^{\Phi }(x)c_1^2, \end{aligned}$$

Similarly for 16, we obtain

20 $$\begin{aligned} -(2-u_2)a_2 = C_1^{\Phi }(x)d_1, \end{aligned}$$

21 $$\begin{aligned} 8 \bigg [2(3-u_3)-u_2(2-u_2)\bigg ]a_2^2-(3-u_3)a_3 = C_1^{\Phi }(x)d_2+C_2^{\Phi }(x)d_1^2. \end{aligned}$$

From Eqs. (18) and (20), we get

22 $$\begin{aligned} c_1 = -d_1, \end{aligned}$$

and

23 $$\begin{aligned} 2(2-u_2)^2a_2^2 = [C_1^{\Phi }(x)]^2[c_1^2+d_1^2]. \end{aligned}$$

Summing up Eqs. (19) and (21), we have

24 $$\begin{aligned} 2\bigg [ (3-u_3)-u_2(2-u_2) \bigg ] a_2^2 = C_1^{\Phi }(x)(c_2+d_2)+C_2^{\Phi }(x)(c_1^2+d_1^2) \end{aligned}$$

By equating Eqs. (22) and (23), we obtain

25 $$\begin{aligned} 2 \bigg [ (3-u_3) - u_2(2-u_2) - \frac{(2-u_2)^2C_2^{ \Phi }(x)}{(C_1^{\Phi }(x))^2}\bigg ]a_2^2 = C_1^{\Phi }(x)(c_2+d_2). \end{aligned}$$

It is well know from Ref.^32^ that if

$$\begin{aligned} |w(\mu )|<1 \ \ and \ \ |v(\nu )|<1, \end{aligned}$$

then

26 $$\begin{aligned} |c_j|\le 1 \ \ and \ \ |d_j|\le 1 \ \ \forall \ \ j\in {\mathcal {N}} \end{aligned}$$

By applying Eqs. (26) and (5) in Eq. (25). We obtain

27 $$\begin{aligned} 2 \bigg | (3-u_3) - u_2(2-u_2) - \frac{(2-u_2)^2[ 2\alpha (1+\alpha )x^2-\alpha ]}{4 \alpha ^2 x^2}\bigg ||a_2^2| \le 4|\alpha | x. \end{aligned}$$

Rearrange the above equation to get the necessary inequality (Eq. 9).

28 $$\begin{aligned} \small {|a_2| \le \frac{2|\Phi |x \sqrt{2|\Phi |x}}{\sqrt{|\Phi (2-u_2)^2- 2\tau x^2|}} } \end{aligned}$$

Next, by subtracting Eq. (21) from Eq. (19), we have

29 $$\begin{aligned} a_3-a_2^2 = \frac{C_1^{\Phi }(x) (c_2-d_2)}{2(3-u_3)}+\frac{C_2^{\Phi }(x)(c_1^2-d_1^2)}{2(3-u_3)} \end{aligned}$$

Further, in view of Eq. (22), it follows from Eq. (26) that

30 $$\begin{aligned} |a_3| = |a_2^2|+\frac{C_2^{\Phi }(x)(|c_2-d_2|)}{2(3-u_3)} \end{aligned}$$

By making use of the Eqs. (23) and (26), we get from aforementioned equation the desired inequality (Eq. 10).

31 $$\begin{aligned} |a_3| \le \frac{4|\Phi ^2| x^2}{(2-u_2)^2}+\frac{2|\Phi |x}{(3-u_3)} \end{aligned}$$

where $$u_n = \frac{1-t^n}{1+t}$$ , $$\tau = \Phi (1+\Phi )(2-u_2)^2-2\Phi ^2 [(3-u_3)-u_2(2-u_2)]$$ $$\square$$

## Proposed methodology

**Fig. 1:**
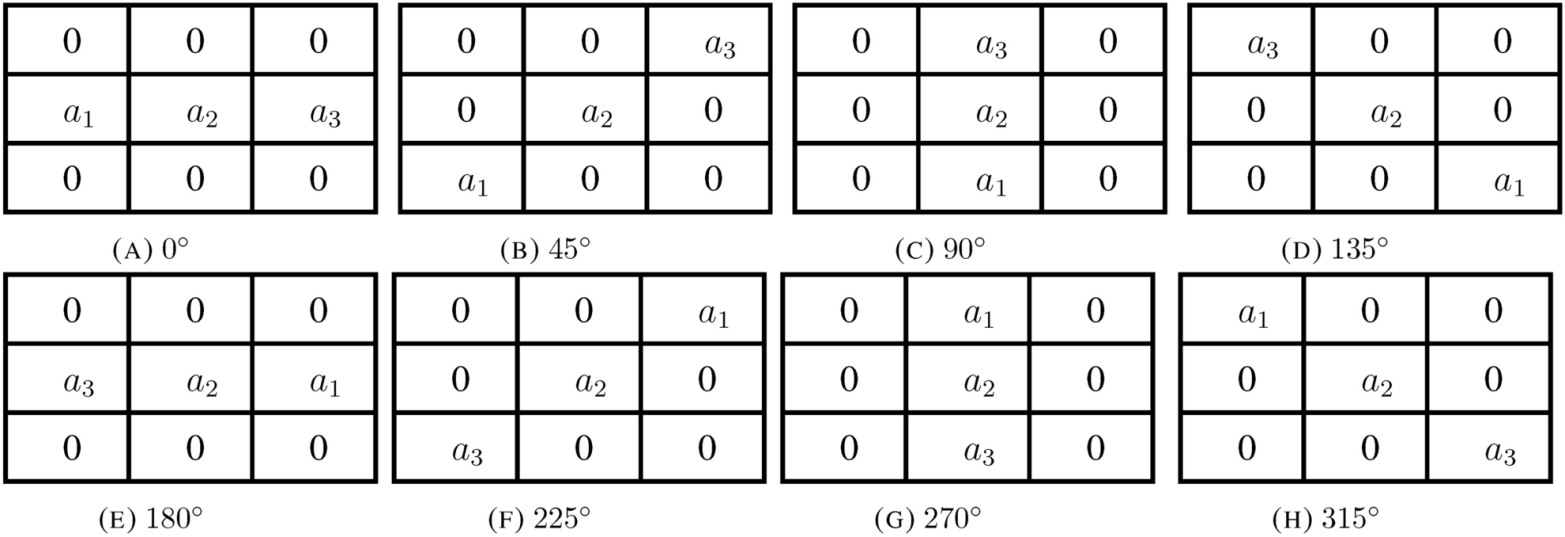
Kernels.

We introduce a new mathematical approach in this study for improving images in low light conditions. Our approach makes use of coefficients obtained from the class $G_S (\Phi )$ to develop a highly effective enhancement technique. The coefficient bounds act as guiding factors, ensuring a controlled and effective enhancement that addresses the challenges posed by low-light environments, ultimately contributing to improved image quality. In image processing, a kernel, also known as a convolution matrix or mask, plays a pivotal role in filtering and transforming images. It is a small matrix composed of coefficient bounds that is convoluted with an input image, enabling operations like blurring, sharpening, embossing, and edge detection. Kernels are essentially mathematical functions that dictate how each pixel in the output image depends on the values of nearby pixels, including the pixel itself. To achieve effective image enhancement with low computational complexity, we have chosen to employ $3 \times 3$ kernels over larger sizes, as increasing the kernel size can lead to image blurring. The $3 \times 3$ kernel size strikes a balance between enhancement and preserving image details. The kernels associated with the coefficients in eight directions are given in Fig 1.

The proposed method is as follows.

- Step 1: Take a low light color image of size $(M\times N)$.
- Step 2: Define the *t* ranges to be $-1$ to 0.9.
- Step 3: Determine the coefficient bounds $|a_1|$ (Eq. 8), $|a_2|$ (Eq. 9) and $|a_3|$ (Eq. 10).
- Step 4: Create $3 \times 3$ kernels for eight different directions ($0^\circ ,45^\circ ,90^\circ ,135^\circ ,180^\circ ,225^\circ ,270^\circ$ and $315^\circ$).
- Step 5: Apply the previously determined coefficient bounds to the kernels.
- Step 6: Convolute the image with each of these kernels separately for all eight directions. Perform this convolution operation on each individual color channel (R, G, and B).
- Step 7: Calculate the average of the results obtained from the convolution operations.
- Step 8 Introduce some additional brightness to the enhanced image by using the function ’imadd’ which performs pixel-wise addition to contribute to an overall improvement in visual quality in the processed image.
- Step 9: Compute the entropy of the enhanced image. According to entropy theory, the more extensive and detailed the information in an image, the greater the entropy (IE)^33^. Entropy computation aids in determining the optimal parameter for effective low-light image enhancement.
- Step 10: Iteratively repeat Step 3 through Step 9 for each *t* value.
- Step 11: Identify the *t* value that results in the maximum entropy value. This particular *t* value represents the optimal parameter for image enhancement.
- Step 12: Finally, display the enhanced image, which was produced using the chosen optimal *t* value.
  For the proposed methodology, the total computational complexity is $$O(n*M*N)$$.
  Where *n*—number of iterations based on the *t* value
  *M* and *N*—dimensions of the image.

## Experimental analysis and results

The experiment was conducted in the following setting. The machine used for this experiment is a computer with a Dual-Intel Core i3 processor running at 1.1 GHz. This computer is equipped with 8 GB of 3733 MHz LPDDR4X RAM and operates on MacOS 13.5. The experimental setup was executed using Matlab online version (Mathworks). LOL^34^ is a collection of 500 image pairs captured in varying lighting conditions, encompassing scenes such as houses, campuses, clubs, and streets. This dataset is designed for enhancing images taken in low-light situations and includes 485 pairs of training and 15 pairs of testing images. In the low-light images, you can observe noise resulting from the image capture process. The majority of these images depict indoor scenes, and all images have been standardized to a resolution of $400 \times 600$ pixels and are saved in Portable Network Graphics (PNG) format.

**Fig. 2:**
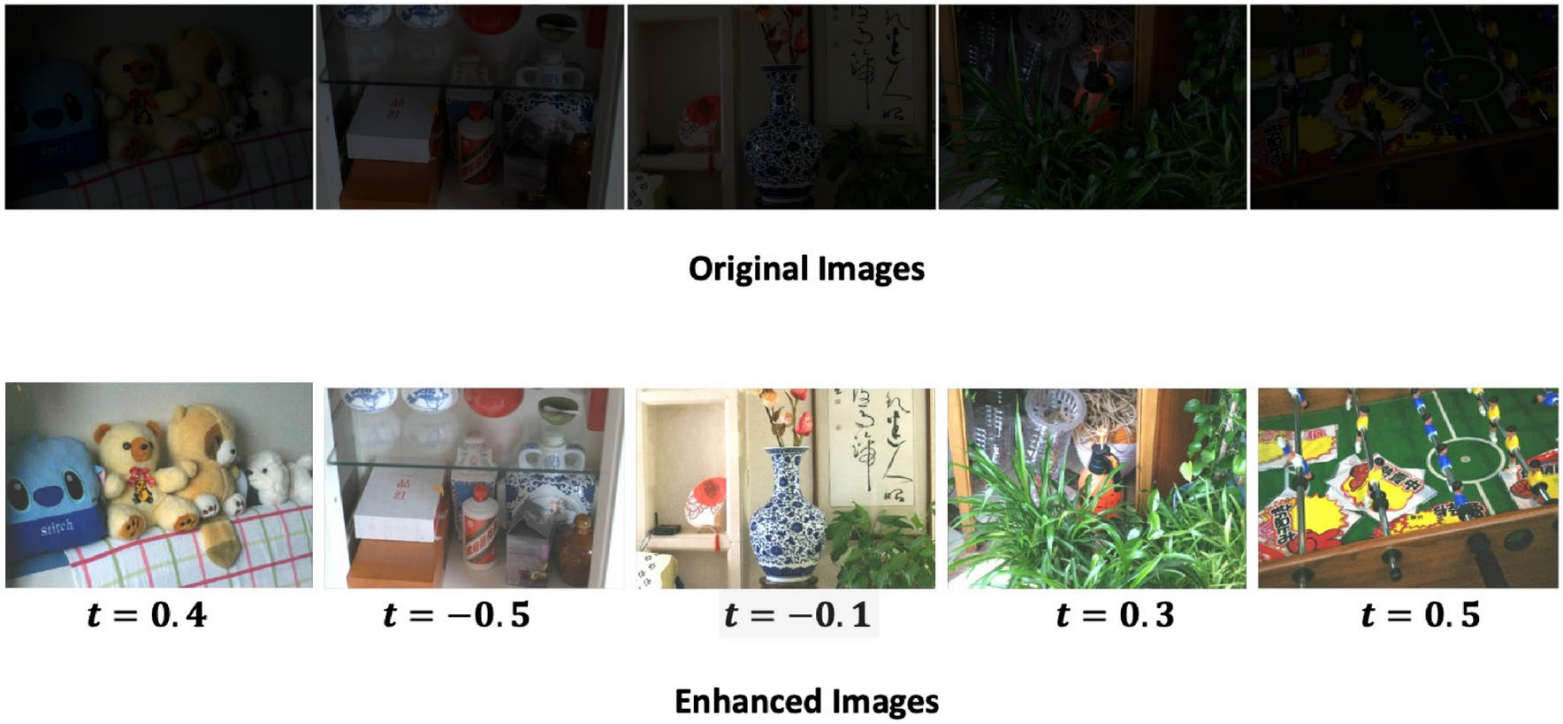
Original images and enhanced images with its corresponding t values.

**Fig. 3:**
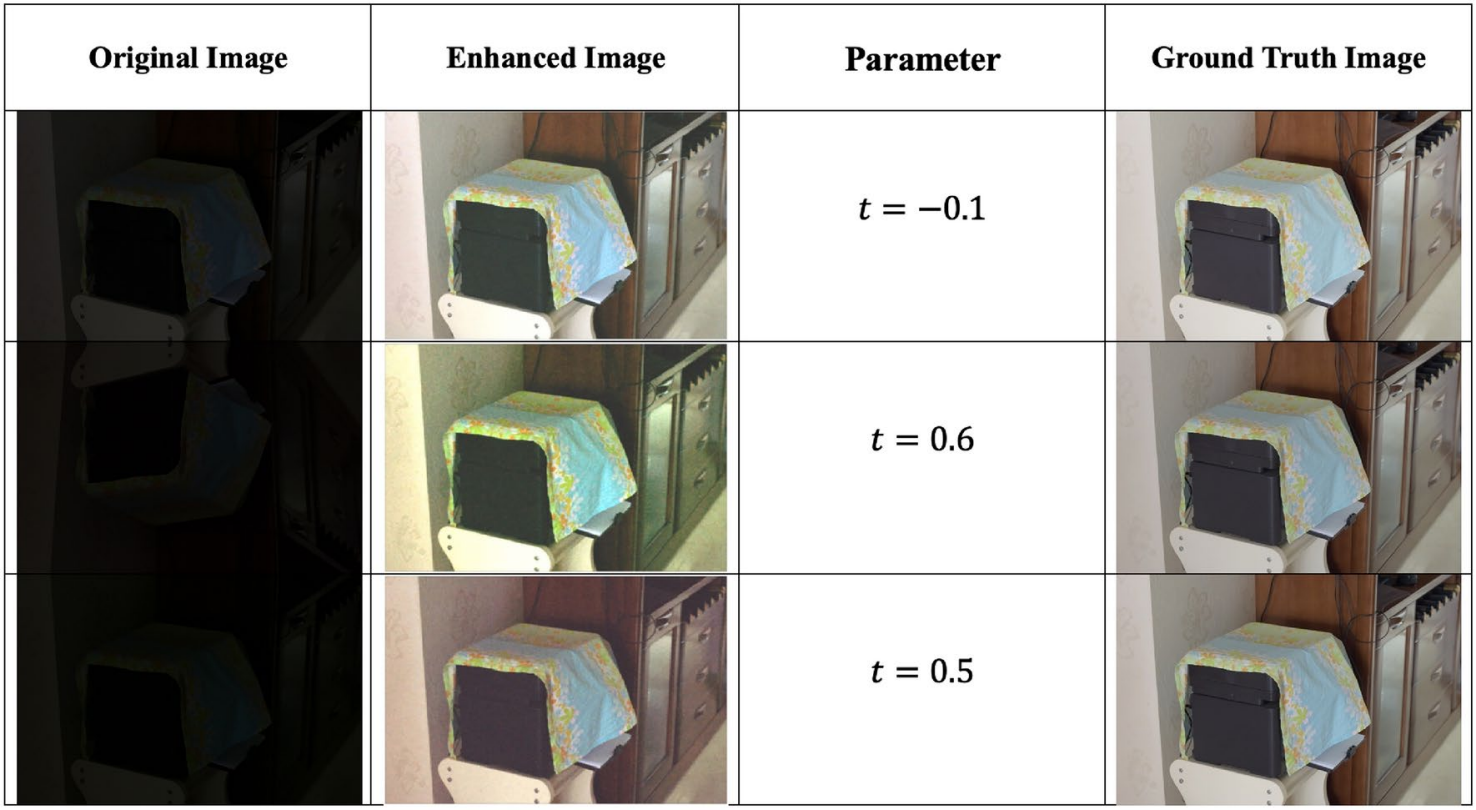
Proposed methods perform across different levels of darkness and noise.

**Fig. 4:**
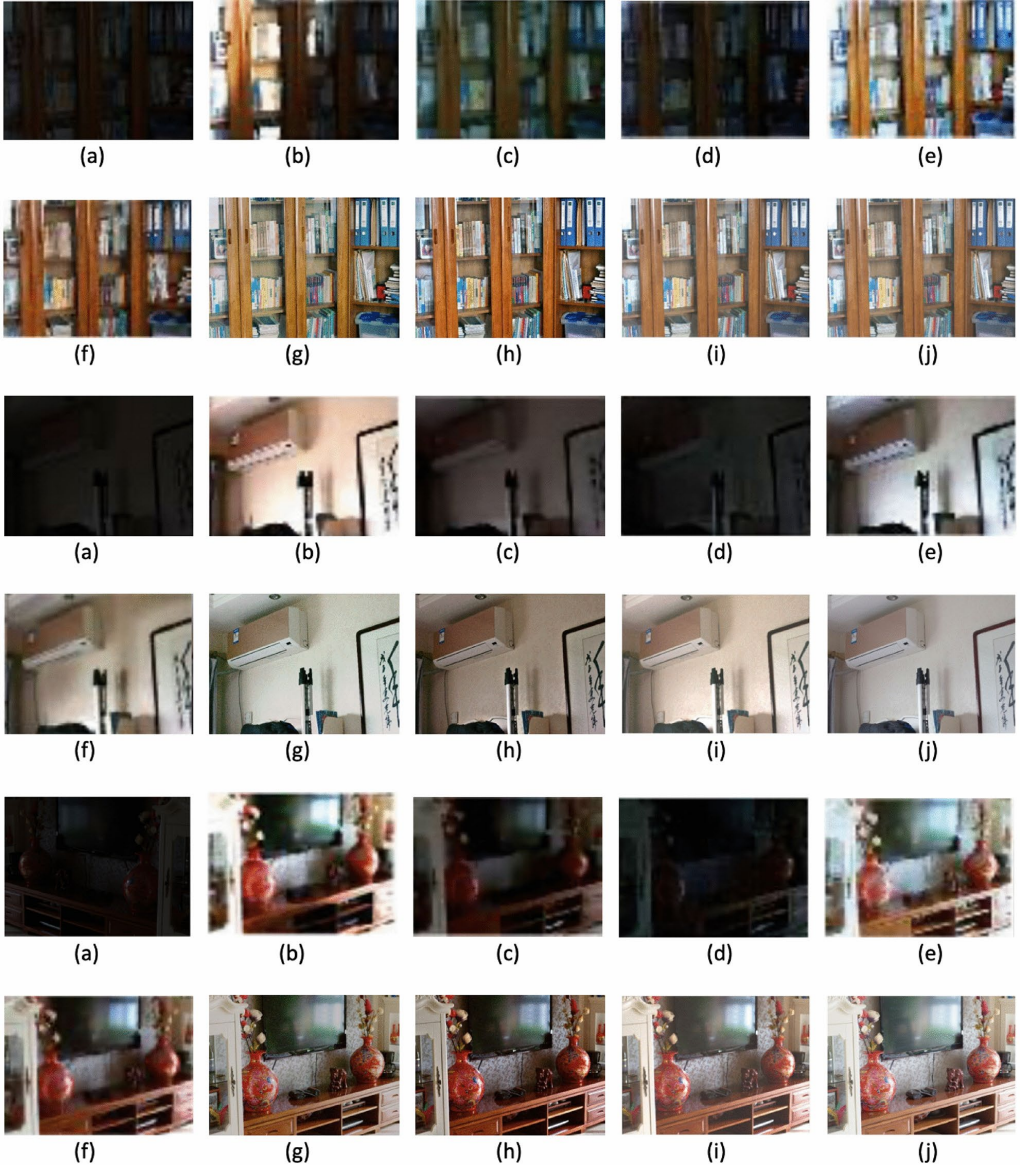
Qualitative comparison of low-light image enhancement methods: (**a**) Input image, (**b**) He et al.^8^, (**c**) Chen et al.^35^, (**d**) Li et al.^36^, (**e**) Ancuti et al.^11^, (**f**) Hari et al.^37^, (**g**) Lecca et al^38^, (**h**) Zhang et al.^39^, (**i**) Our method and (**j**) Ground Truth.

**Fig. 5:**
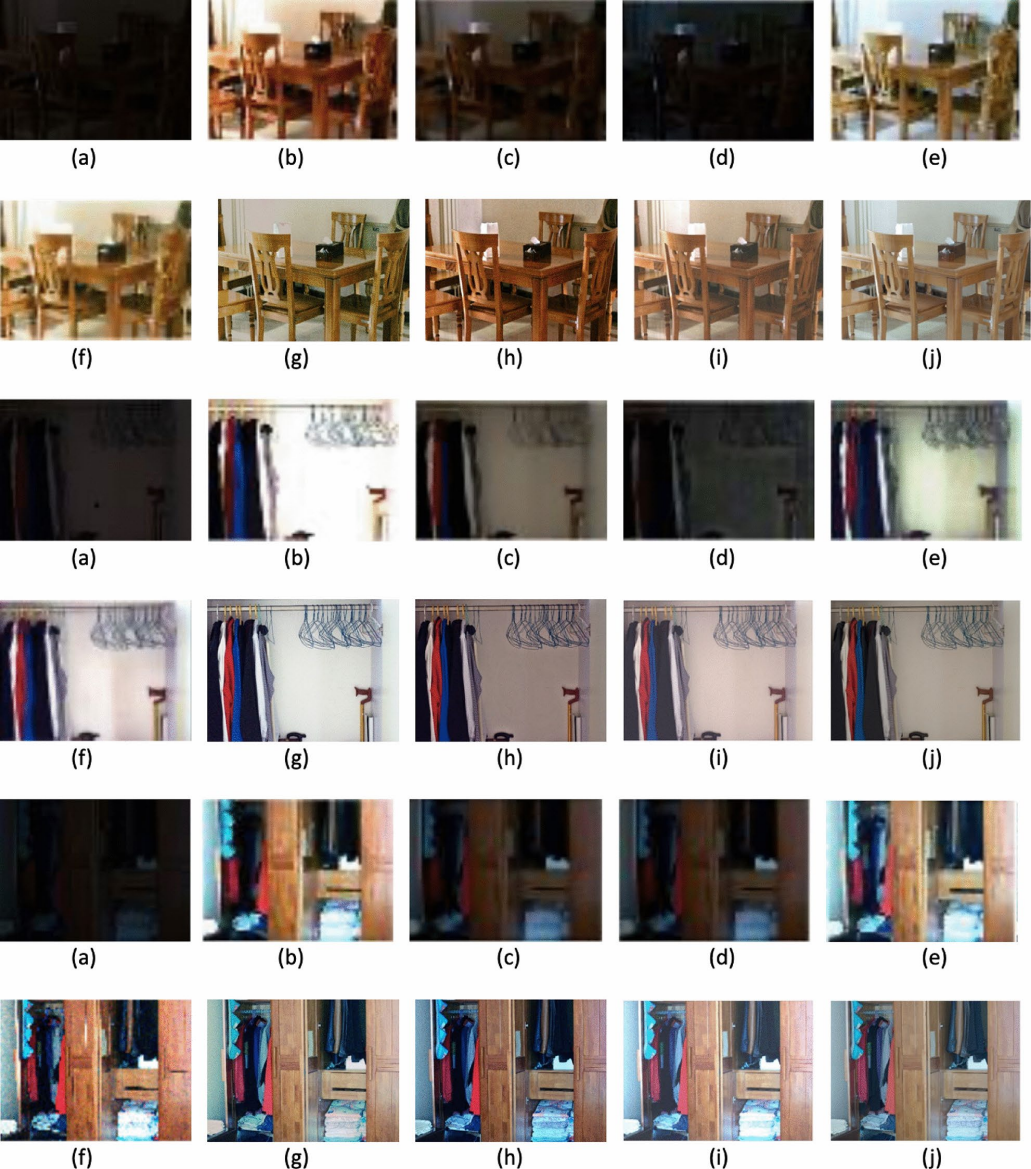
Qualitative comparison of low-light image enhancement methods: (**a**) Input Image, (**b**) He et al.^8^, (**c**) Chen et al.^35^, (**d**) Li et al.^36^, (**e**) Ancuti et al.^11^, (**f**) Hari et al.^37^, (**g**) Lecca et al.^38^, (**h**) Zhang et al.^39^, (**i**) Our method and (**j**) Ground Truth.

**Fig. 6:**
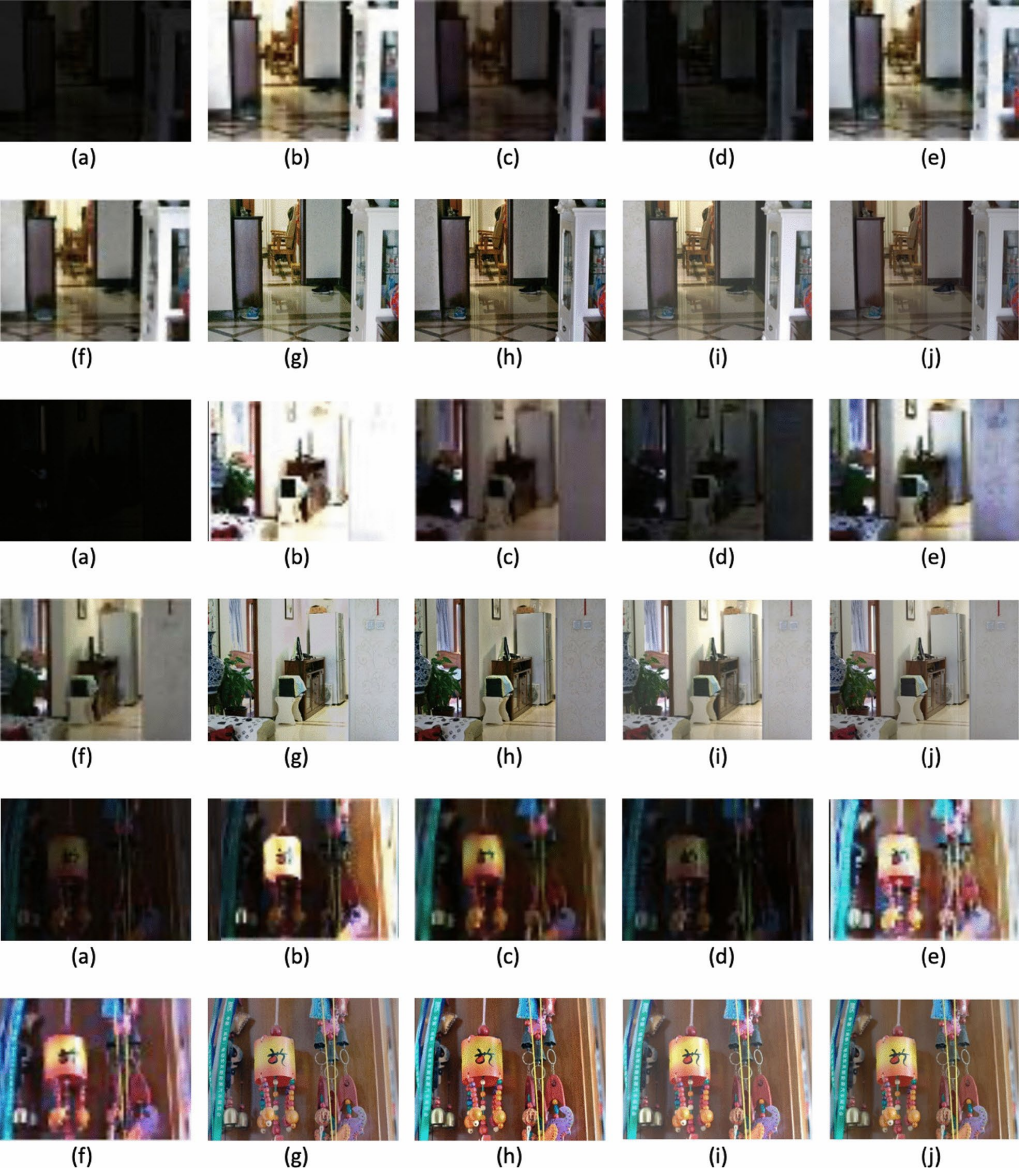
Qualitative comparison of low-light image enhancement methods: (**a**) Input Image, (**b**) He et al.^8^, (**c**) Chen et al.^35^, (**d**) Li et al.^36^, (**e**) Ancuti et al.^11^, (**f**) Hari et al.^37^, (**g**) Lecca et al.^38^, (**h**) Zhang et al.^39^, (**i**) Our method and (**j**) Ground Truth.

Figure 2 presents the original images along with their respective enhanced versions, each associated with its corresponding *t* value. Figure 3 displays sample low-light images sourced from the dataset, highlighting the challenges associated with enhancement. Our method aims to decrease noise levels and generate high-quality images; however, with increasing noise, our approach introduces a subtle color variation in the enhanced image. Figures 4, 5 and 6 displays a clear comparison between our method and other existing techniques. The method proposed by He et al.^8^ generates images with uneven enhancement. Li et al.’s^36^ and Chen et al.’s^35^ method produce images with diminished visibility and limited enhancement. Ancuti et al.’s^11^, Hari et al.’s^37^, Lecca et al.^38^, Zhang et al.^39^ and our method exhibit notable performance on the LoL dataset.

## Performance analysis

Performance-based analysis is a systematic approach for assessing the quality and efficacy of an algorithm or techniques using mathematical functions. It involves the use of specific metrics and criteria to assess how well a particular method performs in achieving its intended objectives. In our study we have considered the quantitative measures such as PSNR, SSIM, and entropy. These analyses provide valuable insights into the strengths and weaknesses of image enhancement techniques.

### PSNR

Peak Signal-to-Noise Ratio (PSNR) serves as a metric for evaluating image quality. It involves the comparison of an image to a reference image. PSNR provides a quantification of how the maximum achievable intensity in a clean image relates to the impact of any noise or errors that might compromise the image’s fidelity.

The formula for PSNR is as follows:

$$\begin{aligned} PSNR = 10 * log_{10} \bigg ( \frac{(P-1)^2}{MSE} \bigg ) \end{aligned}$$

where *P* represents the number of potential intensity levels in the image, with the lowest intensity level considered as 0. The calculation of Mean Squared Error (MSE) in PSNR is defined as:

$$\begin{aligned} MSE = \frac{1}{ij} \sum _{m=0}^{i-1} \sum _{n=0}^{j-1} (O(m,n) - D(m,n))^2 \end{aligned}$$

here *O* signifies the matrix data of the original, noise-free image. *D* stands for the matrix data of the degraded or altered image. *i*, *j* denotes the number of rows and columns of pixels respectively. *m*, *n* signifies the index of a specific row and coloumn respectively.

### SSIM

The Structural Similarity Index Measure (SSIM) is a perceptual image quality metric designed to measure the similarity between an original image and a processed version of it. SSIM considers three fundamental aspects of human visual perception: luminance (brightness), contrast, and structural information. It produces a score within the range of $-1$ to 1, where a score of 1 signifies perfect similarity. A higher SSIM score indicates a greater resemblance between the two images. In our evaluation, we have employed the reference image instead of original image for SSIM calculations. The formula for SSIM is

$$\begin{aligned} SSIM(a, b) = \frac{(2\mu _a\mu _b + C_1)(2\sigma _{ab} + C_2)}{(\mu _a^2 + \mu _b^2 + C_1)(\sigma _a^2 + \sigma _b^2 + C_2)} \end{aligned}$$

where: *a* and *b* are the compared images. $\mu _a$ and $\mu _b$ are the means of *a* and *b*. $\sigma _a^2$ and $\sigma _b^2$ are the variances of *a* and *b*. $\sigma _{ab}$ is the covariance of *a* and *b*. $C_1$ and $C_2$ are constants added to avoid instability when the means and variances are close to zero. These constants are typically small positive values.

### Entropy

Entropy in image processing is a metric that quantifies the amount of information, or randomness, in an image. It is calculated using pixel values and helps evaluate the diversity of intensities within an image. The formula for entropy is

32 $$\begin{aligned} H(X) = - \sum p(x) log_2(p(x)) \end{aligned}$$

where *p*(*x*) represents the probability of a pixel having intensity *x*.

**Fig. 7:**
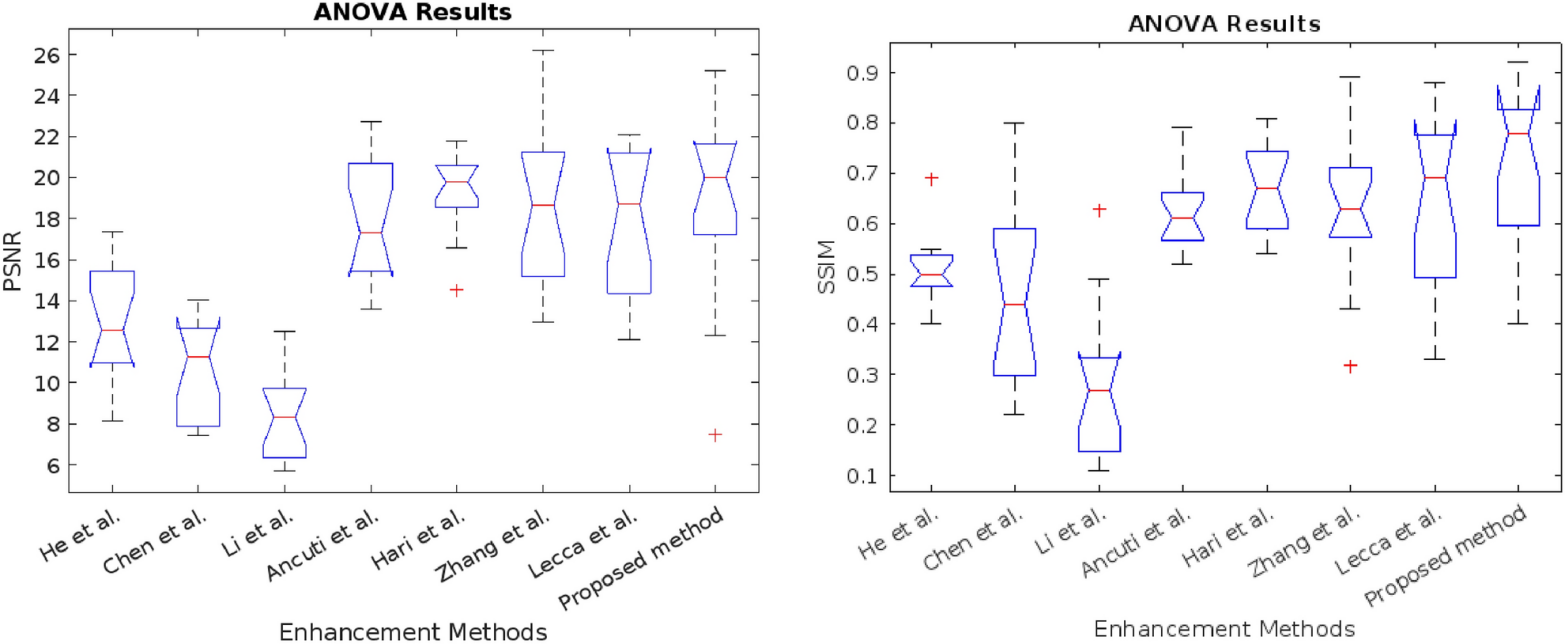
ANOVA test.

**Fig. 8:**
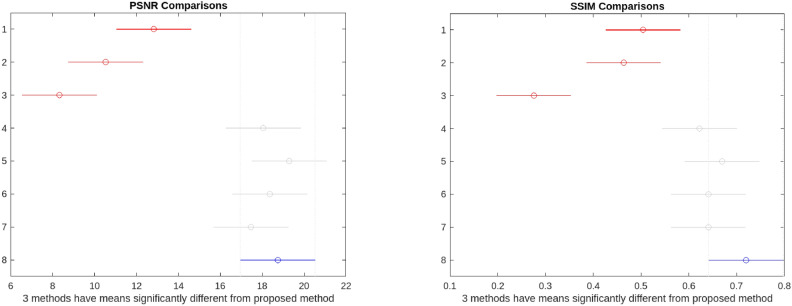
Post Hoc comparison.

Tables 1 and 2 provides a performance comparison of our method with those proposed by He et al.^8^, Chen et al.^35^, Li et al.^36^, Ancuti et al.^11^, Hari et al.^37^, and Lecca et al.^38^ and Zhang et al.^39^ . From the data presented in Tables 1 and 2, it is evident that for the set-15 image, our algorithm outperforms other methods in terms of PSNR and SSIM. Specifically, it performs 46$\%$ better than He et al.^8^, 78$\%$ better than Chen et al.^35^, 125$\%$ better than Li et al.^36^, 4$\%$ better than Ancuti et al.^11^, 2$\%$ better than Zhang et al.^39^ and 7$\%$ better than Lecc et al.^38^. However, it falls slightly behind by 2$\%$ compared to Hari et al^37^. In terms of SSIM, our algorithm demonstrates superiority with 43$\%$ improvement over He et al.^8^, 55$\%$ over Chen et al.^35^, 161 $\%$ over Li et al.^36^, 16$\%$ over Ancuti et al.^11^, 7$\%$ over Hari et al.^8^, 12 $\%$ over Lecca et al.^38^ and Zhang et al.^39^. Although the average PSNR improvement percentage is less than that of Hari et al.^8^, our method preserves similarity structures well and visually produces higher-quality results, even in the presence of noise. We performed a one-way ANOVA to compare the means of the different enhancement methods based on their respective performance metrics. The groups were classified according to the enhancement algorithms, with 15 images taken for each method. The results are presented in the form of a boxplot in Fig. 7. The ANOVA test yielded a p-value of $2.227 \times e^{-21}$ for PSNR comparison and $9.2285 \times e^{-15}$, which is far below the conventional threshold of 0.05. This extremely small p-value indicates that there is a statistically significant difference between the groups. Therefore, we can conclude that at least one of the enhancement methods has a significantly different effect compared to the others. However, to identify which specific methods differ, further post-hoc tests are required to determine whether our method performs significantly better than others. The results from the post hoc analysis revealed in Fig. 8 that the proposed method excels in both PSNR and SSIM metrics, consistently outperforming most of the state-of-the-art techniques tested. With a high average PSNR (mean = 18.7400) and the highest SSIM (mean = 0.7193), our method shows superior effectiveness in enhancing low-light images, preserving both image clarity and structural similarity. The significant differences observed in the statistical analysis further reinforce the robustness of our approach.

**Table 1:** PSNR analysis of the proposed method and state-of-art approaches.

Image no.	He et al.^8^	Chen et al.^35^	Li et al.^36^	Ancuti et al.^11^	Hari et al.^37^	Lecca et al.^38^	Zhang et al.^39^	Proposed
5	10.68	11.49	8.31	19.85	19.14	23.71	20.65	25.22
14	16.47	7.42	5.70	15.26	18.48	21.44	14.81	21.08
35	15.49	8.30	6.59	22.13	21.57	15.06	12.11	20.01
56	15.94	7.98	6.44	22.73	21.76	18.9	12.52	21.88
64	15.28	7.87	6.11	19.75	20.18	18.67	14.63	22.13
145	9.64	13.68	8.85	16.65	16.59	13.66	21.96	17.14
191	17.35	9.87	7.41	19.31	20.05	21.84	18.68	17.37
239	12.56	13.67	9.77	15.35	19.78	16.25	22.06	17.01
243	8.27	12.75	12.50	13.58	18.69	19.1	21.37	20.20
258	12.24	12.29	9.14	17.30	20.67	26.2	22.07	20.09
487	12.86	7.64	6.13	20.99	20.38	15.77	14.4	17.74
538	13.57	7.51	6.31	21.58	21.47	18.2	14.34	21.82
590	8.15	12.14	10.68	14.88	14.54	13.6	13.98	7.50
738	11.85	14.02	11.51	15.61	19.23	12.94	19.63	12.31
782	12.16	11.28	9.57	15.83	16.87	20.54	18.75	19.60

**Table 2:** SSIM analysis of the proposed method and state-of-art approaches.

Image no.	He et al.^8^	Chen et al.^35^	Li et al.^36^	Ancuti et al.^11^	Hari et al.^37^	Lecca et al.^38^	Zhang et al.^39^	Proposed
5	0.4719	0.5587	0.3231	0.7501	0.7457	0.86	0.84	0.9224
14	0.4926	0.4411	0.2212	0.5236	0.5368	0.61	0.48	0.5654
35	0.5479	0.2823	0.1417	0.6917	0.6837	0.71	0.59	0.7758
56	0.4312	0.2778	0.1129	0.5890	0.5784	0.43	0.33	0.5638
64	0.4934	0.3469	0.1421	0.6032	0.6241	0.63	0.5	0.6816
145	0.6904	0.6509	0.3418	0.5241	0.6423	0.55	0.83	0.8293
191	0.5472	0.3849	0.1987	0.6349	0.6662	0.78	0.77	0.8218
239	0.5346	0.5972	0.2750	0.6266	0.6568	0.63	0.66	0.7774
243	0.5430	0.8035	0.4927	0.5549	0.7668	0.71	0.78	0.8554
258	0.5024	0.5378	0.2777	0.6346	0.7811	0.89	0.88	0.9106
487	0.5026	0.2172	0.1725	0.5582	0.5427	0.32	0.33	0.3938
538	0.4059	0.2526	0.1388	0.5864	0.5792	0.58	0.49	0.6762
590	0.4918	0.8038	0.6258	0.7875	0.8147	0.66	0.75	0.4861
738	0.5088	0.4464	0.4012	0.6141	0.7017	0.57	0.69	0.7003
782	0.3965	0.3492	0.2682	0.6674	0.7245	0.69	0.69	0.8109

## Conclusion

The main focus of our work is to explore the application of geometric function theory in the enhancement of low-light images, aiming to achieve a superior enhanced version. The proposed method successfully delivers an improved image with reduced noise, balanced brightness, enhanced clarity, and preservation of crucial details. When compared with other state-of-the-art methods, both qualitative and quantitative assessments demonstrate the superior performance of our approach. In future work, the methodology employed in this study can be extended to video enhancement by applying the same techniques to each frame of the video sequence. This extension involves adapting the low-light image enhancement approach to accommodate the temporal dynamics and characteristics inherent in video data. By consistently applying the proposed methodology across video frames, it becomes possible to enhance the visual quality of entire video sequence, addressing challenges posed by low-light conditions in dynamic scenarios. This expansion would contribute to advancing the applicability of the proposed method in scenarios where video data is crucial, such as surveillance, monitoring, and various computer vision applications where enhancing scenes under such conditions can greatly enhance the accuracy of object detection and recognition.

## Data Availability

The dataset analysed during the current study is “https://daooshee.github.io/BMVC2018website/” The MATLAB code used in this investigation are accessible through the following link: K, Sivagami Sundari; B, Srutha Keerthi (2024), “Low light image enhancement - GFT”, Mendeley Data, V1, doi: 10.17632/2gcrkjr88z.1.
